# Short telomere length and its correlation with gene mutations in myelodysplastic syndrome

**DOI:** 10.1186/s13045-016-0287-9

**Published:** 2016-07-28

**Authors:** Sang Mee Hwang, Seon Young Kim, Jung Ah Kim, Hee-Sue Park, Si Nae Park, Kyongok Im, Kwantae Kim, Sung-Min Kim, Dong Soon Lee

**Affiliations:** 1 Department of Laboratory Medicine, Seoul National University Bundang Hospital, Seongnam, Republic of Korea; 2 Department of Laboratory Medicine, Seoul National University College of Medicine, Seoul, Republic of Korea; 3 Department of Laboratory Medicine, Chungnam National University College of Medicine, Daejeon, Republic of Korea; 4 Cancer Research Institute, Seoul National University College of Medicine, Seoul, Republic of Korea

**Keywords:** Telomere length, Quantitative fluorescence in situ hybridization, Single cell, Mutation

## Abstract

**Background:**

Telomere erosion can lead to genomic instability and cancer progression. It has been suggested that the shortest telomere, not the average telomere length (TL), is critical for cell viability. Some studies have shown shorter TL in myelodysplastic syndrome (MDS) patients but the critically short telomeres, the variability of TL within individual patient has not been evaluated. Thus, we aimed to investigate the TL of MDS patients and assessed the association of TL with recurrent genetic mutations in MDS.

**Methods:**

We measured the TL of bone marrow nucleated cells for diagnostic samples at a single-cell level by quantitative fluorescence in situ hybridization (Q-FISH) for 58 MDS patients and analyzed the minimum, median, average, standard deviation, average of the 0th to 10th percentile TL within a patient, and the proportion of cells with TL that is shorter than the lowest 10th percentile of the normal control (NC). The correlations of TL to clinical parameters, cytogenetic results, and genetic mutations were assessed.

**Results:**

MDS patients showed eroded telomeres and narrow distribution compared to the NC (*P* < 0.001, *P* = 0.018, respectively). Patients with mutation showed significantly lesser cells with short TL, below the lowest 10th percentile of the NC (*P* = 0.017), but no differences in TL were found according to mutations/cytogenetic abnormalities except for *CSF3R* mutation. However, those patients with a high percentage (≥80 %) of cells with short TL showed poorer overall survival (*P* = 0.021), and this was an independent prognostic factor, along with *TP53*, *U2AF1* mutation, and high BM blast count (*P* = 0.044, 0.001, 0.004, 0.012, respectively).

**Conclusions:**

The shortest TL, which determines the fate of the cell, was significantly shorter, and higher burden of cells with short TL were found in MDS, which correlated with poor survival, suggesting the need to measure TL in single cells by Q-FISH.

**Electronic supplementary material:**

The online version of this article (doi:10.1186/s13045-016-0287-9) contains supplementary material, which is available to authorized users.

## Background

Telomeres are non-coding, repetitive sequences of DNA at the ends of the chromosomes of eukaryotic cells which become shorter as cells divide, and when telomere attrition reaches its limit, cell proliferation arrest, senescence, and apoptosis can occur [1, 2]. Shortened telomeres can cause end-to-end fusion of chromosomes, which results in genomic instability and contributes to carcinogenesis [3]. Thus, telomeres and their maintenance have been studied in many hematologic malignancies [4–6].

Myelodysplastic syndromes (MDS) are clonal hematopoietic stem cell diseases characterized by cytopenia, dysplasia, and ineffective hematopoiesis, with an increased risk of transformation to acute myeloid leukemia (AML) [7, 8]. Due to its predisposition to AML, genomic instability has been studied as the key to the pathogenesis of MDS, and thus, many studies have assessed the telomere components of MDS [9–11]. MDS patients have shown a shorter average telomere length (TL) compared to normal control (NC), but the association of TL to other clinical parameters in patients with MDS has been inconsistent [9, 11, 12].

Many somatic mutations have been identified in patients with MDS, involving various pathways including epigenetic regulation, RNA splicing machinery, and transcription factors [13–15], which help elucidate the pathophysiology of the disease. A recent study showed that telomere dysfunction causes aberrant RNA splicing by repressing the splicing gene expression [16]. However, the correlation of TL with various genetic mutations other than telomerase complex genes has not been thoroughly explored [17, 18].

Moreover, most previous studies have used Southern blot analysis, quantitative PCR, or flow fluorescence in situ hybridization (FISH) for the analysis of TL [9, 11, 19, 20]. However, these studies only assessed the mean or median TL of the patient whereas it is believed that the cell with the *shortest* telomere is actually the critical factor leading telomere dysfunction [3, 21]. Performing quantitative-FISH (Q-FISH) for telomeres allows the measurement of TL at a single-cell level with the measurement of the distribution of TL of within individuals as well as the shortest TL [22]. Telomeres of individual chromosomes are stained with peptic nucleic acid probes for telomeres, and the fluorescence intensity of the telomeric signal is measured with fluorescence-based automatic microscope [23]. The fluorescence intensity is normalized by the use of an additional centromeric probe, and the telomere to centromere (T/C) fluorescence intensity ratio is considered to be the surrogate of TL since the fluorescence intensity of the telomeric signal is known to be proportional to the TL [24]. Inclusion of the centromeric probe also allows the selection of single cells in which two centromeric signals are observed and the normalization of the signal for hybridization [23]. Performing Q-FISH in interphase nuclei further allows measurement of each value in numerous nucleated cells. Therefore, we measured the TL of bone marrow (BM) nucleated cells of patients with MDS at diagnosis by Q-FISH, evaluated the correlation of the TL with genetic mutations and clinical parameters, and explored the usefulness of TL parameters as a prognostic marker.

## Methods

### Patients

This study examined 58 patients diagnosed with MDS (37 men and 21 women) at Seoul National University Hospital between January 2004 and December 2014. This study was approved by the institutional review board of Seoul National University Hospital (1604-082-754). The samples included in this study were from diagnostic samples of MDS. The characteristics of the MDS patients are shown in Table 1. BM aspirates from 23 individuals who underwent marrow biopsy and showed no evidence of hematologic malignancy were included as the NC. The bone marrow of the NC showed no evidence of bone marrow involvement and showed normal karyotype.

**Table 1:** Characteristics of MDS patients (*n* = 58)

Characteristics	Number (%)
Gender (Male/female)	37: 21
Median age (year, range)	69 (18–86)
Age (years)	
<60	22 (37.9)
≥60	36 (62.1)
Complete blood count at bone marrow sample collection	
White blood cell (10^3^/μL)	2.6 (0.6–17.2)
Hemoglobin (g/dL)	8.0 (4.4–12.2)
Absolute neutrophil count (/μL)	1151 (80–11,345)
Platelet count (10^3^/μL)	74.5 (3–1029)
Median bone marrow blast (%, range)	2.3 (0.0–18.1)
WHO category	
RCUD	12 (20.7)
RARS	4 (6.9)
RCMD	13 (22.4)
RAEB	20 (34.5)
MDS, U	9 (15.5)
IPSS risk groups	
Low	8 (13.8)
Intermediate-1	28 (48.3)
Intermediate-2	15 (25.9)
High	7 (12.1)
Revised IPSS	
Very low	4 (6.9)
Low	14 (24.1)
Intermediate	14 (24.1)
High	14 (24.1)
Very High	12 (20.7)
Cytogenetic results	
Karyotype	
Abnormal karyotype	32 (55.2)
Complex karyotype (>3 abnormalities)	9 (15.5)
del(5q)/ –5	6 (10.5)
del(7q)/ –7	8 (14.0)
Trisomy 8	8 (14.0)
Abnormal 17p	3 (5.2)
del(20q)	5 (8.8)
Fluorescence in situ hybridization	
Abnormal chromosome 1^a^	5 (11.1)
Abnormal chromosome 5^b^	8 (17.4)
Abnormal chromosome 7^b^	9 (19.6)
Abnormal chromosome 8^b^	7 (15.2)
Abnormal chromosome 20^b^	4 (8.7)

*RCUD* refractory cytopenia of unilineage dysplasia, *RCMD* refractory cytopenia of multilineage dysplasia, *MDS*, *U* myelodysplastic syndrome, unclassifiable, *IPSS* International Prognostic Scoring System

^a^Only performed in 45 patients

^b^Performed in 46 patients

### Karyotyping—Giemsa banding

A standard protocol was used to perform Giemsa banding with heparinized whole blood marrow samples [25]. A minimum of 20 metaphase cells were karyotyped according to the International System for Human Cytogenetic Nomenclature (ISCN) [26, 27].

### Interphase fluorescence in situ hybridization for MDS

Interphase FISH tests (del(5q)/−5, del(7q)/−7, trisomy 8, del(20q), trisomy 1/1q+) were performed. The probes used were Vysis LSI EGR1 (5q31)/D5S23, D5S721 Dual color probe kit, Vysis LSI D7S522 (7q31)/CEP7 FISH probe, Vysis CEP8(Dz82) Spectrum Green Probe, Vysis D20S108 FISH probe, and Vysis LSI 1p36/LSI1q25 and LSI 19q13/19p13 Dual color probe (Abbott Molecular, Downers Grove, IL, USA). These five types of FISH items were selected based on the previous report of recurrent cytogenetic changes in Korean patients with MDS [28]. FISH was performed with mononuclear cells of BM aspirates at the time of BM examination as described in a previous study [28].

### Telomere quantitative fluorescence in situ hybridization

**Fig. 1:**
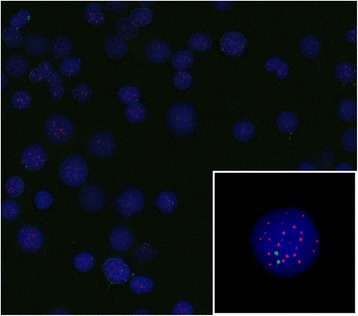
Interphase telomere fluorescence in situ hybridization (FISH) image. FISH results obtained with pan-telomere (cyanine (Cy)3-labeled) and centromere 2 specific (fluorescein isothiocyanate (FITC)-labeled) peptic nucleic acid probe in interphase nuclei. Counter stain was performed with 4′,6-diamidino-2-phenylindole (DAPI) to identify the nuclei

Cryopreserved BM nucleated cells in fixative after FISH preparation were used for telomere analysis. Q-FISH was performed using a Cy3-labelled telomere PNA (peptide nucleic acid) FISH kit (DakoCytomation Denmark A/S, Glostrup, Denmark) and a FITC-labeled PNA probe for the centromere of chromosome 2 (kindly provided by DakoCytomation). One microliter of the probe for the centromere of chromosome 2 was added to 10 μL of the telomere probe. Telomere and centromere Q-FISH hybridizations were performed according to the manufacturer’s instructions. Interphase Q-FISH images were captured with a Zeiss Axioplan 2 imaging microscope (Carl Zeiss MicroImaging GmbH, Munchen, Germany) equipped with ISIS software (MetaSystems GmbH, Altlussheim, Germany) (Fig. 1). For TL measurements, the ISIS-Telomere module (MetaSystems) was used as described previously [23]. The software calculates a T/C fluorescence intensity ratio, which is a measure of TL, for individual cells. At least 25 interphase nuclei were scanned for each patient to measure the TL. The cell with the shortest TL is represented as the minimum TL and the lower 25th percentile value of each individual is expressed as the “Q1” TL. The median, the average and the distribution of TL, standard distribution (SD), and the average of the 0th to 10th percentile TL for each individual was calculated from the total cells of which the TL was measured. Furthermore, the percentage of cells with TL shorter than the 10th percentile of the normal control was assessed and stated as “short” TL.

### Targeted sequencing

Eighty-seven genes were selected for targeted sequencing (Additional file 1: Table S1). Genes that were previously reported as mutational targets in MDS (*n* = 49) [13, 14, 29] or those mutated in other myeloid and lymphoid hematologic malignancies (*n* = 11 and *n* = 27) were included [13, 14, 29–35]. The BM aspirates collected in a sodium ethylenediaminetetraacetic acid tube were treated with ammonium chloride for RBC lysis, and the cell pellets were kept in RNAlater solution (Qiagen, Valencia, CA, USA) at −80 °C until genetic analysis. The genomic DNA (gDNA) was extracted from the buffy coat of BM aspirate using QIAamp DNA Blood Mini Kit (Qiagen), according to the manufacturer’s instructions. The gDNA shearing, standard library production, and hybridization were performed by Celemics Inc. (Seoul, Korea). The quality of the gDNA was assessed by the Agilent 2200 TapeStation System (Agilent, Santa Clara, CA, USA). A total target length of the 259-kb region using the paired-end 150-bp rapid-run sequencing mode was performed on an Illumina Hiseq 2500 platform (Illumina, San Diego, CA, USA). The sequencing data calling process is described in Additional file 1.

### Statistical analysis

Categorization of MDS patients was performed according to the 2008 World Health Organization (WHO) classification of tumors of hematopoietic and lymphoid tissues [8], the International Prognostic Scoring System (IPSS), and the revised IPSS et al. [36, 37]. The correlation of TL with various clinical parameters, BM findings, cytogenetic abnormalities, and mutations found in multi-gene panels was assessed.

The gene mutations were evaluated separately and in subgroups according to the function of genes. Ten gene subgroups are splicing machinery, DNA methylation, chromatin modification, transcription factor, receptor/kinases, RAS pathway, cell signaling, DNA repair/cell cycle, cohesion, and miscellaneous. The genes in each subgroup are listed in the Additional file 1: Table S1. The *χ*2 test and Fisher’s exact test were used to compare categorical variables. Pairwise correlations between TLs and gene mutations, cytogenetic results, and categorical clinical parameters were evaluated using Kendall tau-b (*T*_b_) correlation. For linear parameters, Pearson’s *r* correlation was used. The comparisons of TLs between patients with and without a certain mutation were made with the Mann-Whitney method for genes that showed mutation in more than 5.0 % of the patients.

The overall survival (OS) was calculated from the date of diagnosis to the date of death from any cause and compared by Kaplan-Meier method (log-rank test). Univariable and multivariable Cox analyses were performed to assess variables as prognostic factors for survival. Backward LR selection was performed for multivariable Cox analysis. Harrell’s C-index was calculated for the proposed multivariable Cox model and the revised IPSS. Statistical analyses were performed using the SPSS version 22.0 (IBM Inc., Chicago, IL, USA) and *R* statistical program (http://www.r-project.org). *P* values <0.05 were considered to be statistically significant.

## Results

### Telomere lengths and clinical profiles

We compared the telomere lengths (TLs) of the MDS patients (*n* = 58) with those of the NC (*n* = 23), according to several parameters (Table 2). The clinical characteristics of the MDS patients included in this study are shown in Table 1. There was no significant difference in age and sex (*P* = 0.175, 0.796, respectively) between the MDS patients and NC. However, the number of cells assessed in the MDS patients was smaller (*P* = 0.004) than that assessed in the NC due to insufficient nuclei in the patient samples. All of the TL parameters were significantly lower in the MDS patients than the NC (*P* < 0.001 for all the TL except SD, *P* = 0.018 for SD). The distribution of the telomere length of each individual was narrower in the MDS patients than the NC (*P* = 0.018).

**Table 2:** Telomere length parameters of MDS patients and normal control

Parameters	Average ± SD		*P* value
	MDS (*n* = 58)	NC (*n* = 23)	
Age	63.7 ± 14.6	60.9 ± 9.5	0.175
Gender (M/F)	37:21	16:7	0.796
No. of cells assessed for telomere lengths	77.6 ± 38.9	98.1 ± 26.3	0.004
Telomere lengths (T/C ratio)			
Minimum	1.97 ± 1.47	4.41 ± 1.36	<0.001
25th percentile (Q1)	5.49 ± 3.04	11.79 ± 3.06	<0.001
Median	8.56 ± 4.45	15.92 ± 3.93	<0.001
Average	10.08 ± 4.73	17.29 ± 4.42	<0.001
Average of 0–10 percentile	2.95 ± 1.76	6.64 ± 1.74	<0.001
Standard Deviation	6.67 ± 3.01	8.07 ± 2.73	0.018
Cells under the 10th percentile of the NC (%)	52.1 ± 24.9		

*MDS* myelodysplastic syndrome, *NC* normal control

We also calculated the lowest 10th percentile TL value of the NC group and found out that 52.1 (±24.9)% of MDS patients’ cells were *under* this length (Table 2). These results show that not only the mean TL of the MDS patients is shorter than that of the NC but also a large proportion of MDS patients’ cells are shorter than the lowest 10th percentile TL of the NC.

**Fig. 2:**
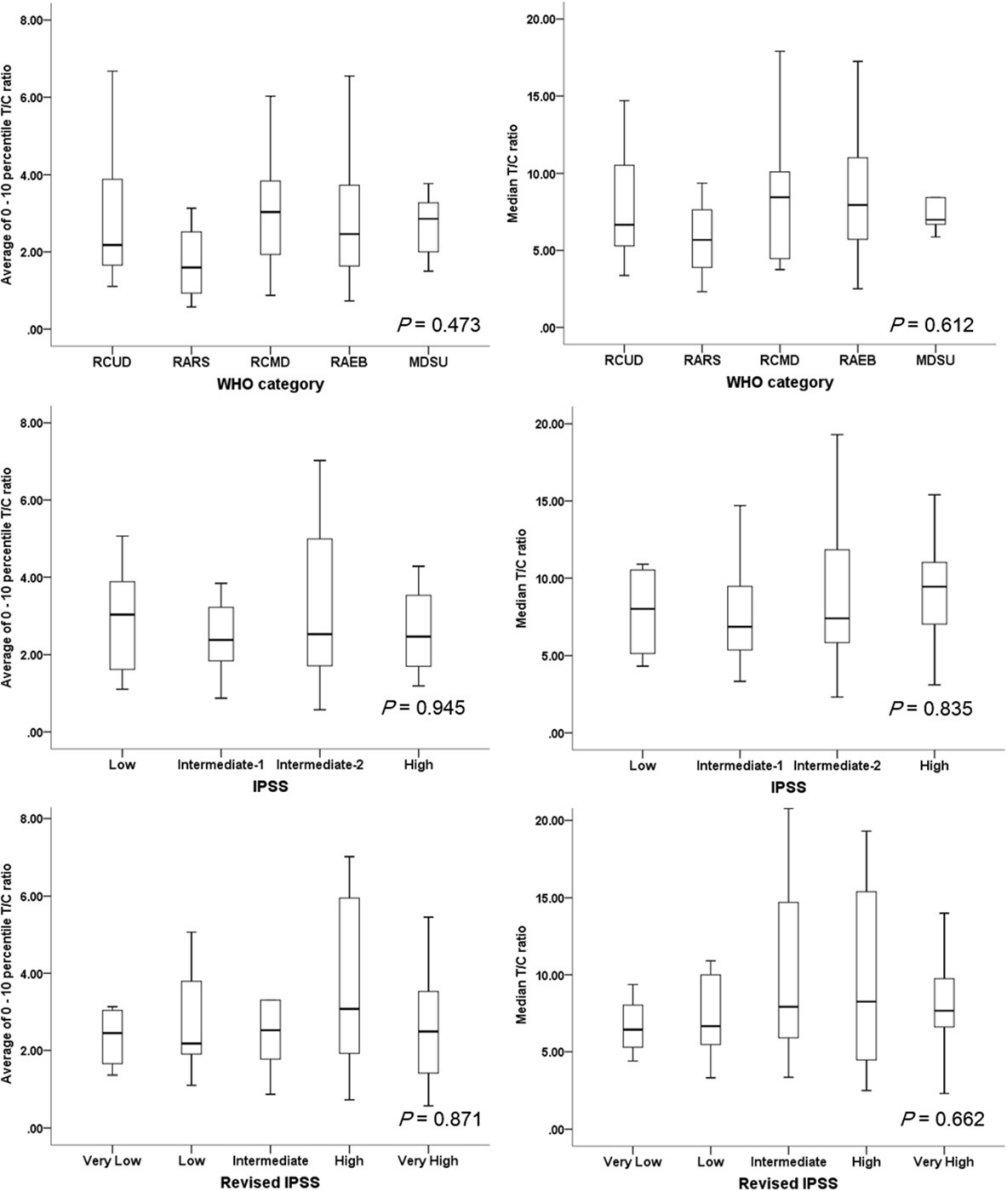
Telomere lengths according to the World Health Organization (WHO) categories, International Prognostic Scoring System (IPSS), and revised IPSS subgroups. *T/C* telomere to centromere

TL was compared for WHO categories and IPSS risk groups. The TL parameters were not different among the clinical subgroups (the average of the 0th–10th percentile TL and the median TL shown in Fig. 2, the other TL parameters are in the Additional file 1: Table S2). Additional comparison of TL was made according to the complete blood count and BM findings. Patients with low hemoglobin (<8 g/dL) showed a higher percentage of cells with TL less than the 10th percentile of normal control (*P* = 0.020). However, the other TL parameters were not significantly different in those patients with hemoglobin values less than vs. equal to/greater than 8 g/dL. There were no significant differences in the TL according to absolute neutrophil count, platelet count, BM blast count, fibrosis, or cellularity (Additional file 1: Table S3).

### Multi-gene panel results

**Fig. 3:**
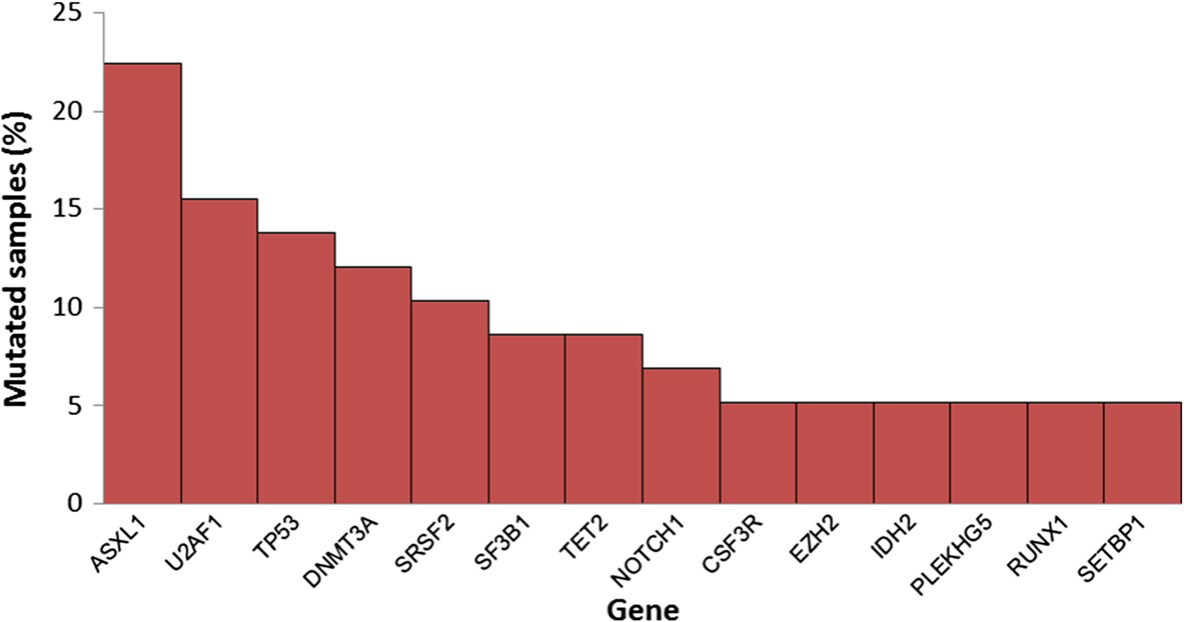
The percentage of samples that was found with specific gene mutations in genes with mutations in more than 5 % of the patients

**Fig. 4:**
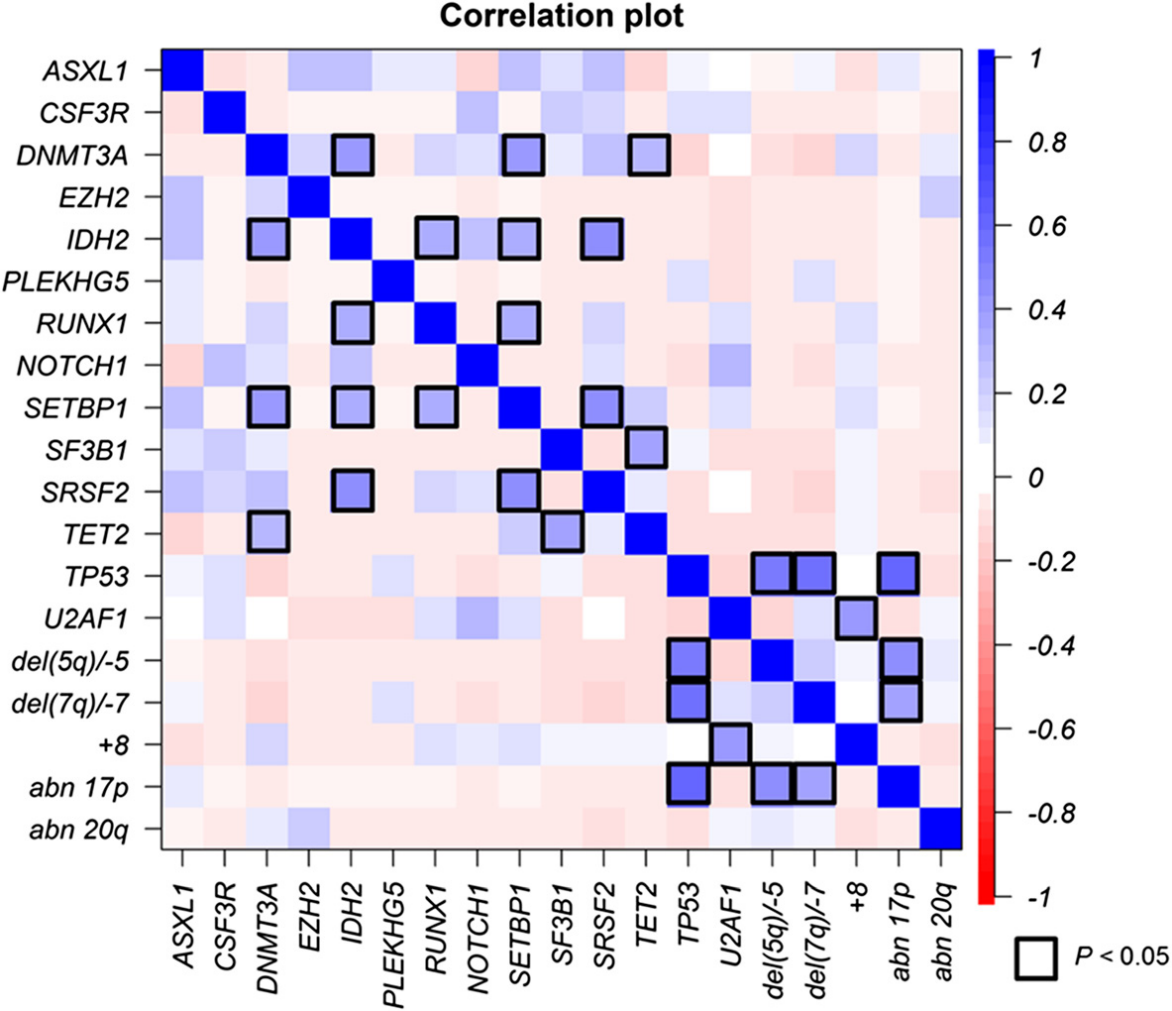
Correlations between gene mutations and cytogenetic changes. Significant correlations are marked with *bold border*

Among the 87 genes, we found mutations of 43 genes in more than one patient. Forty-five patients (77.6 %) had showed mutation in one or more of the genes. Only the gene mutations found in more than 5 % of the samples are shown in Fig. 3. Grouping the 87 genes by gene function, genes related to splicing were most frequently mutated (34.5 %), following the genes for transcription factors (31.0 %), chromatin modification (25.9 %), DNA methylation (19.0 %), receptor/kinases (15.5 %), and RAS pathways (8.6 %). The associations of gene mutations and/or cytogenetic results are shown in Fig. 4. Kendall tau-b (*T*_b_) correlation coefficients greater than 0.40 were present between *SRSF2* and *SETBP1* mutation (*T*_b_ = 0.432, *P* = 0.001), *IDH2* and *SRSF2* mutation (*T*_b_ = 0.432, *P* = 0.001), *TP53* mutation and del(5q)/−5 (*T*_b_ = 0.520, *P* < 0.001), *TP53* mutation and del(7q)/−7 (*T*_b_ = 0.564, *P* < 0.001), *TP53* mutation and abnormalities of 17p (*T*_b_ = 0.583, *P* < 0.001), del(5q)/−5 and abnormality of 17p (*T*_b_ = 0.431, *P* = 0.001). Significant correlations were also present between *DNMT3A* vs. *IDH2* mutation, *DNMT3A* vs. *SETBP1* mutation (*T*_b_ = 0.390, *P* = 0.003, respectively), *U2AF1* mutation vs. trisomy 8 (*T*_b_ = 0.379, *P* = 0.005), del(7q)/−7 vs. abnormal 17p (*T*_b_ = 0.357, *P* = 0.008), *SF3B1* vs. *TET2* mutation (*T*_b_ = 0.343, *P* = 0.010), *DNMT3A* vs. *TET2* mutation (*T*_b_ = 0.263, *P* = 0.047), *IDH2* vs. *RUNX1* mutation, *RUNX1* vs. *SETBP1* mutation, *IDH2* vs. *SETBP1* mutation (*T*_b_ = 0.297, *P* = 0.025, respectively). Comparing the association between gene categories and cytogenetic abnormalities; DNA methylation vs. cell signaling-related genes (*T*_b_ = 0.391, *P* = 0.003), DNA methylation vs. DNA repair/cell cycle-related genes (*T*_b_ = 0.389, *P* = 0.003), RAS pathway vs. splicing-related genes (*T*_b_ = 0.294, *P* = 0.026), splicing-related genes vs. trisomy 8 (*T*_b_ = 0.338, *P* = 0.011), transcription factor genes vs. del(5q)/−5 (*T*_b_ = 0.276, *P* = 0.039), transcription factor genes vs. del(7q)/−7 (*T*_b_ = 0.399, *P* = 0.003), transcription factor genes vs. abnormality of 17p (*T*_b_ = 0.362, *P* = 0.007) were shown to have significant correlations (Additional file 1: Figure S1).

### Telomere length and genetic mutations/cytogenetic abnormalities

We analyzed whether somatic mutations had a relationship with TL. Table 3 shows the TL of patients according to whether or not they have a certain somatic mutation. Patients without gene mutations had shorter TL than those with mutation. All of the TL parameters showed significant differences (minimum, Q1, median, average, average of 0–10 percentile, SD, percentage of cells under the 10th percentile of the NC) between those patients with or without gene mutations (*P* = 0.043, 0.041, 0.025, 0.014, 0.030, 0.007, 0.017, respectively). Looking at specific mutations, the difference in TL was only present for those with or without *CSF3R* mutation. Patients without *CSF3R* mutation (*n* = 55) had a significantly higher percentage of cells whose length was below the lowest 10th percentile of the NC than those with *CSF3R* mutation (*n* = 3) (*P* = 0.037). In addition, comparisons were made for the TL according to gene groups. However, there were no significant differences in TL parameters by 10 different gene groups (Additional file 1: Table S4). Since TL varies according to age in the normal population, the age was compared between the mutational status of the genes or gene groups. Significant differences were only found for *TET2* mutational status and the presence/absence of mutation in DNA methylation genes category. Patients with *TET2* mutation and mutations of DNA methylation were older than those without mutation (*P* = 0.021, 0.008, respectively). The TL was not significantly different between patients with or without certain cytogenetic abnormalities by karyotyping or FISH (Table 3). In summary, we found that the presence of any gene mutation, but not the cytogenetic results, was related to TL.

**Table 3:** Telomere lengths according to gene mutations or cytogenetic changes

Gene or cytogenetic changes		Number	Mean age	*P* value	Telomere lengths (T/C ratio)													
					Minimum	*P* value	Q1	*P* value	Median	*P* value	Average	*P* value	Average of 0–10 percentile	*P* value	Standard deviation	*P* value	Cells under the 10th percentile of normal control (%)	*P* value
Any gene mutation	Mut(+)	45	65.4 ± 12.2	0.356	2.14 ± 1.53	0.043	5.82 ± 3.07	0.041	8.99 ± 4.17	0.025	10.68 ± 4.56	0.014	3.18 ± 1.82	0.030	7.12 ± 2.97	0.007	48.2 ± 23.6	0.017
	Mut(−)	13	57.9 ± 20.6		1.38 ± 1.11		4.34 ± 2.80		7.12 ± 5.26		8.00 ± 4.90		2.14 ± 1.32		5.13 ± 2.74		65.8 ± 25.5	
*ASXL1*	Mut(+)	13	65.4 ± 10.8	0.837	2.13 ± 2.29	0.461	5.32 ± 3.79	0.557	8.04 ± 4.83	0.595	9.39 ± 5.20	0.450	2.97 ± 2.28	0.689	5.78 ± 2.40	0.310	57.4 ± 24.4	0.396
	Mut(−)	45	63.2 ± 15.7		1.92 ± 1.17		5.54 ± 2.85		8.72 ± 4.39		10.28 ± 4.63		2.94 ± 1.62		6.93 ± 3.15		50.6 ± 25.1	
*CSF3R*	Mut(+)	3	57.3 ± 21.1	0.438	2.21 ± 0.99	0.438	7.87 ± 2.87	0.087	12.43 ± 3.71	0.062	14.23 ± 4.18	0.067	3.82 ± 2.29	0.459	9.23 ± 2.43	0.119	23.9 ± 13.8	0.037
	Mut(−)	55	64.0 ± 14.4		1.96 ± 1.50		5.36 ± 3.03		8.36 ± 4.42		9.86 ± 4.69		2.90 ± 1.74		6.53 ± 3.00		53.7 ± 24.5	
*DNMT3A*	Mut(+)	7	72.0 ± 8.9	0.117	1.66 ± 0.77	1.000	4.38 ± 1.25	0.439	6.77 ± 1.58	0.425	8.14 ± 1.66	0.453	2.50 ± 0.75	0.907	5.43 ± 1.88	0.779	55.6 ± 15.4	0.312
	Mut(−)	51	62.6 ± 15.0		2.01 ± 1.55		5.65 ± 3.20		8.82 ± 4.67		10.35 ± 4.96		3.01 ± 1.86		6.84 ± 3.12		51.7 ± 26.0	
*EZH2*	Mut(+)	3	71.7 ± 0.6	0.240	3.95 ± 3.95	0.379	7.71 ± 5.74	0.547	10.66 ± 6.28	0.525	12.57 ± 7.71	0.502	4.44 ± 3.43	0.342	7.13 ± 3.97	0.869	62.3 ± 12.5	0.594
	Mut(−)	55	63.3 ± 14.9		1.86 ± 1.22		5.37 ± 2.88		8.45 ± 4.39		9.95 ± 4.59		2.87 ± 1.65		6.65 ± 3.00		51.6 ± 25.4	
*IDH2*	Mut(+)	3	66.7 ± 9.1	0.843	3.08 ± 2.20	0.232	6.18 ± 4.53	0.974	10.04 ± 6.42	0.715	11.70 ± 5.50	0.570	3.91 ± 2.73	0.525	8.05 ± 0.99	0.232	38.7 ± 29.1	0.525
	Mut(−)	55	63.5 ± 14.9		1.91 ± 1.43		5.46 ± 3.00		8.49 ± 4.39		9.99 ± 4.73		2.90 ± 1.72		6.60 ± 3.08		52.9 ± 24.8	
*NOTCH1*	Mut(+)	4	64.0 ± 17.1	0.824	2.47 ± 0.84	0.189	8.01 ± 4.97	0.348	11.94 ± 7.12	0.363	14.43 ± 7.72	0.236	4.24 ± 2.29	0.248	9.48 ± 3.33	0.067	35.3 ± 31.7	0.332
	Mut(−)	54	63.7 ± 14.6		1.93 ± 1.51		5.31 ± 2.84		8.32 ± 4.19		9.76 ± 4.38		2.85 ± 1.71		6.46 ± 2.92		53.4 ± 24.2	
*PLEKHG5*	Mut(+)	3	58.7 ± 10.3	0.324	1.01 ± 0.52	0.158	2.86 ± 2.05	0.111	5.07 ± 2.45	0.138	6.99 ± 3.37	0.291	1.57 ± 1.17	0.119	5.95 ± 2.90	0.843	70.3 ± 18.0	0.193
	Mut(−)	55	64.0 ± 14.9		2.02 ± 1.49		5.64 ± 3.04		8.76 ± 4.47		10.25 ± 4.76		3.02 ± 1.77		6.71 ± 3.04		51.1 ± 25.0	
*RUNX1*	Mut(+)	3	56.7 ± 10.6	0.193	2.13 ± 1.27	0.690	4.90 ± 1.76	0.843	10.16 ± 5.47	0.641	11.73 ± 5.44	0.570	2.83 ± 0.92	0.766	8.91 ± 4.08	0.193	58.0 ± 14.9	0.641
	Mut(−)	55	64.1 ± 14.8		1.96 ± 1.49		5.53 ± 3.11		8.48 ± 4.44		9.99 ± 4.73		2.95 ± 1.80		6.55 ± 2.95		51.8 ± 25.4	
*SETBP1*	Mut(+)	3	66.7 ± 20.0	0.895	1.76 ± 0.32	0.690	4.20 ± 1.02	0.547	6.48 ± 1.80	0.594	7.61 ± 2.03	0.459	2.41 ± 0.43	0.869	4.76 ± 2.01	0.246	58.6 ± 18.7	0.690
	Mut(−)	55	63.5 ± 14.5		1.98 ± 1.51		5.56 ± 3.11		8.68 ± 4.54		10.22 ± 4.81		2.98 ± 1.81		6.78 ± 3.04		51.8 ± 25.3	
*SF3B1*	Mut(+)	5	63.6 ± 11.2	0.788	2.09 ± 1.60	0.872	5.13 ± 1.68	0.747	7.99 ± 2.51	0.851	9.87 ± 3.23	0.768	2.72 ± 1.48	0.893	7.79 ± 3.99	0.553	47.2 ± 19.2	0.517
	Mut(−)	53	63.7 ± 15.0		1.96 ± 1.48		5.53 ± 3.16		8.62 ± 4.61		10.10 ± 4.87		2.97 ± 1.80		6.57 ± 2.94		52.6 ± 25.5	
*SRSF2*	Mut(+)	6	73.8 ± 8.0	0.101	2.40 ± 1.72	0.446	6.47 ± 3.76	0.558	10.10 ± 5.48	0.446	11.42 ± 5.68	0.508	3.70 ± 2.26	0.374	6.89 ± 2.55	0.737	42.4 ± 30.5	0.416
	Mut(−)	52	62.5 ± 14.8		1.92 ± 1.45		5.38 ± 2.98		8.39 ± 4.35		9.93 ± 4.65		2.86 ± 1.70		6.65 ± 3.09		53.2 ± 24.3	
*TET2*	Mut(+)	5	75.8 ± 6.1	0.021	2.85 ± 1.69	0.131	7.06 ± 2.70	0.086	10.06 ± 2.89	0.162	12.11 ± 3.22	0.138	3.91 ± 1.91	0.154	8.19 ± 3.81	0.316	33.4 ± 18.5	0.076
	Mut(−)	53	62.6 ± 14.7		1.89 ± 1.44		5.35 ± 3.06		8.43 ± 4.57		9.89 ± 4.83		2.86 ± 1.74		6.53 ± 2.94		53.9 ± 24.8	
*TP53*	Mut(+)	8	69.0 ± 8.9	0.471	1.81 ± 1.23	0.748	5.12 ± 3.06	0.974	8.44 ± 4.08	0.748	10.25 ± 4.36	0.650	2.67 ± 1.84	0.471	7.62 ± 3.05	0.260	47.2 ± 27.0	0.471
	Mut(−)	50	62.8 ± 15.3		1.99 ± 1.52		5.55 ± 3.07		8.59 ± 4.55		10.05 ± 4.83		2.99 ± 1.77		6.52 ± 3.01		52.9 ± 24.7	
*U2AF1*	Mut(+)	9	59.4 ± 12.7	0.179	2.02 ± 1.01	0.408	6.62 ± 3.49	0.225	10.39 ± 5.60	0.269	12.08 ± 6.35	0.318	3.49 ± 1.69	0.201	7.77 ± 4.21	0.512	47.6 ± 27.7	0.614
	Mut(−)	49	64.5 ± 15.0		1.96 ± 1.55		5.29 ± 2.95		8.23 ± 4.20		9.71 ± 4.36		2.85 ± 1.78		6.47 ± 2.75		53.0 ± 24.6	
Karyotype	Normal	25	61.2 ± 15.1	0.416	2.00 ± 1.95	0.237	5.32 ± 3.44	0.385	7.95 ± 4.46	0.274	9.36 ± 4.93	0.222	2.93 ± 2.09	0.459	6.08 ± 3.01	0.216	57.7 ± 26.6	0.123
	Abnormal	32	65.3 ± 14.3		1.92 ± 1.02		5.64 ± 2.80		9.00 ± 4.53		10.53 ± 4.61		2.94 ± 1.53		7.00 ± 2.96		48.3 ± 23.3	
del(5q)/−5	(+)	6	68.7 ± 13.3	0.393	2.18 ± 1.20	0.500	6.71 ± 3.56	0.393	10.28 ± 4.58	0.233	12.33 ± 4.40	0.177	3.78 ± 2.16	0.352	8.39 ± 2.36	0.048	35.4 ± 28.4	0.089
	(−)	51	62.9 ± 14.9		1.93 ± 1.52		5.35 ± 3.02		8.34 ± 4.48		9.75 ± 4.76		2.84 ± 1.73		6.39 ± 3.00		54.4 ± 24.1	
del(7q)/−7	(+)	8	61.5 ± 18.6	0.866	2.13 ± 1.05	0.302	6.58 ± 3.00	0.111	10.44 ± 5.45	0.175	11.59 ± 4.90	0.207	3.25 ± 1.51	0.261	7.06 ± 2.96	0.449	39.8 ± 24.2	0.096
	(−)	49	63.8 ± 14.2		1.93 ± 1.55		5.32 ± 3.08		8.23 ± 4.30		9.76 ± 4.73		2.89 ± 1.83		6.52 ± 3.02		54.5 ± 24.80	
Trisomy 8	(+)	8	54.4 ± 20.5	0.123	1.87 ± 1.02	0.778	6.35 ± 3.84	0.628	11.09 ± 6.69	0.261	12.35 ± 6.63	0.291	2.95 ± 1.64	0.813	8.29 ± 4.26	0.302	51.3 ± 27.8	0.919
	(−)	49	65.0 ± 13.2		1.97 ± 1.56		5.36 ± 2.96		8.12 ± 3.97		9.64 ± 4.34		2.94 ± 1.82		6.32 ± 2.69		52.6 ± 24.8	
Abn 17p	(+)	3	72.3 ± 10.1	0.307	2.72 ± 1.47	0.324	6.05 ± 3.84	0.749	8.89 ± 5.48	0.906	10.34 ± 5.00	0.880	3.72 ± 2.24	0.599	6.25 ± 1.63	0.801	45.4 ± 33.9	0.623
	(−)	54	63.0 ± 14.8		1.91 ± 1.49		5.47 ± 3.07		8.52 ± 4.49		10.00 ± 4.78		2.90 ± 1.77		6.62 ± 3.06		52.8 ± 24.8	
del(20q)	(+)	5	68.0 ± 10.7	0.632	2.16 ± 0.55	0.186	5.56 ± 1.41	0.519	8.13 ± 2.15	0.795	9.70 ± 2.27	0.712	3.13 ± 0.73	0.262	6.12 ± 1.28	0.880	44.4 ± 15.0	0.327
	(−)	52	63.1 ± 15.0		1.94 ± 1.55		5.49 ± 3.20		8.58 ± 4.66		10.05 ± 4.94		2.92 ± 1.85		6.65 ± 3.11		53.2 ± 25.8	
FISH 1	(+)	5	54.0 ± 25.2	0.428	1.61 ± 1.04	0.562	7.03 ± 4.89	0.586	11.77 ± 7.84	0.368	13.19 ± 7.37	0.349	2.92 ± 2.09	0.903	8.99 ± 3.72	0.128	40.4 ± 29.4	0.314
	(−)	40	63.0 ± 13.9		1.89 ± 1.18		5.49 ± 3.20		8.28 ± 3.98		9.71 ± 4.24		2.86 ± 1.64		6.34 ± 2.72		51.9 ± 25.4	
FISH 5	(+)	8	67.3 ± 9.8	0.485	1.80 ± 1.20	0.765	5.60 ± 3.63	0.921	9.17 ± 4.77	0.559	10.97 ± 4.87	0.450	2.92 ± 2.23	0.579	7.86 ± 2.91	0.123	43.4 ± 30.7	0.338
	(−)	38	61.3 ± 16.2		1.85 ± 1.15		5.45 ± 2.86		8.55 ± 4.52		9.88 ± 4.64		2.87 ± 1.54		6.33 ± 2.87		52.1 ± 24.5	
FISH 7	(+)	9	69.3 ± 7.0	0.160	1.94 ± 1.08	0.605	5.57 ± 2.66	0.478	8.54 ± 3.65	0.643	10.08 ± 4.10	0.723	2.92 ± 1.63	0.870	6.69 ± 2.74	0.786	44.4 ± 15.04	0.461
	(−)	37	60.6 ± 16.4		1.82 ± 1.18		5.46 ± 3.06		8.68 ± 4.75		10.07 ± 4.83		2.87 ± 1.67		6.58 ± 2.98		53.2 ± 25.8	
FISH 8	(+)	7	59.6 ± 15.5	0.490	1.90 ± 1.10	0.858	5.77 ± 3.74	1.000	9.70 ± 5.85	0.697	11.43 ± 6.59	0.697	3.01 ± 1.76	0.881	7.90 ± 4.44	0.549	55.6 ± 27.2	0.549
	(−)	39	62.8 ± 15.5		1.83 ± 1.17		5.43 ± 2.86		8.47 ± 4.30		9.82 ± 4.29		2.85 ± 1.65		6.37 ± 2.55		49.7 ± 25.5	
FISH 20	(+)	4	64.3 ± 7.6	0.985	2.37 ± 1.03	0.173	5.82 ± 1.49	0.417	8.38 ± 2.22	0.721	9.85 ± 2.20	0.721	3.35 ± 1.06	0.229	6.02 ± 1.08	0.895	44.0 ± 15.3	0.510
	(−)	42	62.0 ± 15.8		1.78 ± 1.17		5.39 ± 3.10		8.60 ± 4.74		10.00 ± 4.89		2.79 ± 1.70		6.62 ± 3.06		52.0 ± 26.7	

### Survival analysis

**Fig. 5:**
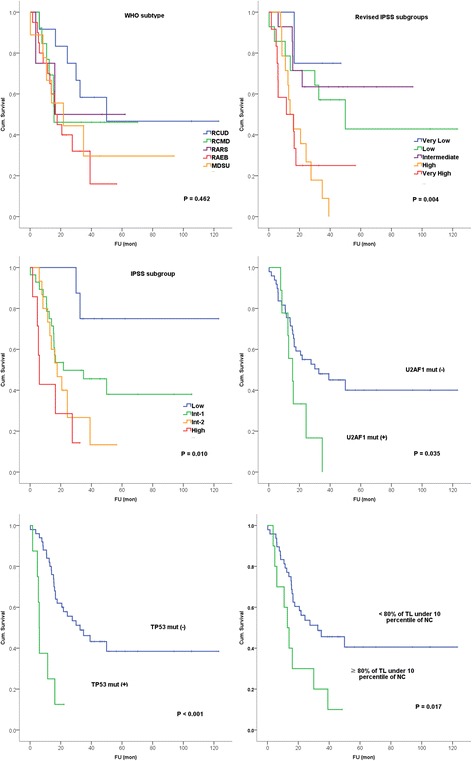
Kaplan-Meier survival curves according to WHO subtypes, IPSS subgroups, telomere lengths, and *TP53* and *U2AF1* mutation status

The OS was compared among different clinical subgroups (Fig. 5). There were significant differences in survival among different IPSS and the revised IPSS risk groups (*P* = 0.010, 0.004, respectively). However, no difference in OS was observed among the WHO categories (*P* = 0.462) by Kaplan-Meier survival analysis.

The OS between those with or without certain gene mutations was compared in genes with mutations that were found in more than 5 % of the patients. We found that patients with *TP53* mutation and *U2AF1* mutation had a significantly shorter OS compared to those without mutation (median survival 32.6 vs. 6.0 months, *P* < 0.001 for *TP53* mutation, 32.6 vs. 15.5, *P* = 0.035 for *U2AF1* mutation). We also compared the OS of patients with ≥80 vs. <80 % of their cells with TL below the lowest 10th percentile TL of normal controls, and the former was significantly shorter (12.9 vs. 32.6 months, *P* = 0.017). The OS was significantly different in patients with ≥80 vs. <80 % of their cells with TL below the lowest 10th percentile TL of the NC even within WHO subtypes and IPSS subgroups (*P* = 0.038, 0.022, respectively). The presence of other mutations was not related to the OS.

Patients with complex karyotype (*P* = 0.015), del(7q)/−7 by karyotype (*P* < 0.001), abnormalities of 17p by karyotype (P = 0.001), abnormalities on FISH for chromosome 5 and 7 (*P* = 0.014, *P* < 0.001, respectively), high BM blast count (≥10 %) (*P* = 0.014), and those patients with ≥80 % of TL under the 10th percentile of the NC (*P* = 0.021) showed significantly lower survival than those without abnormalities. Univariable and multivariable Cox analyses for overall survival were performed with the clinical parameters, mutational profiles and TL parameters (Table 4). Excluding those parameters with significant correlation and many missing values, multivariable Cox analysis showed that the *TP53* mutation, *U2AF1* mutation, high BM blast count (≥10 %), and high percentage (≥80 %) of cells with TL less than the 10th percentile of the NC were independent prognostic factors for survival in patients with MDS (Table 4). The Harrell’s C-index was 0.649 (0.579–0.719) for the revised IPSS only and 0.717 (0.654–0.779) for the model including TL, *TP53*, and *U2AF1* mutation and blast count showing similar prediction power for survival in our model including TL.

**Table 4:** Univariable and multivariable Cox analysis of the overall survival of 58 MDS patients

	Univariable			Multivariable		
Risk factors	HR	95 % CI	*P*	HR	95 % CI	*P*
Revised IPSS			0.010			
Very low vs. low	2.242	0.275–18.287	0.451			
Very low vs. intermediate	1.577	0.184–13.552	0.678			
Very low vs. high	6.731	0.874–51.865	0.067			
Very low vs. very high	6.432	0.808–51.197	0.079			
Complex CG vs. not	2.915	1.229–6.918	0.015			
del(7q)/−7 vs. normal (karyotype)	4.483	1.951–10.302	<0.001			
FISH 5 abnormal vs. normal	2.994	1.248–7.183	0.014			
BM blast ≥10 vs. <10 %	2.476	1.200–5.112	0.014	2.623	1.237–5.563	0.012
*TP53* mutated vs. normal	4.875	2.031–11.703	<0.001	5.257	1.341–33.095	0.001
*U2AF1* mutated vs. normal	2.325	1.035–5.222	0.041	3.544	1.485–8.458	0.004
Percentage of cells with low TL compared to NC ≥80 vs. <80 %	2.47	1.148–5.314	0.021	2.248	1.023 – 4.937	0.044

## Discussion

The findings of shortened TL in many MDS patients have led to the studies of telomere dynamics [9, 11, 38, 39] and its relationship with MDS [16]. The shortest TL in an individual, which is considered to be critical in cellular survival and telomere dysfunction [21, 40] was measured by Q-FISH in a single cell level in patients with MDS. We have found out that in addition to the mean TL, the minimum was also shorter in the MDS patients, and the patients showed a narrower range of TL than the NC. In average, 52.2 % of each patient had a TL of lower than the 10th percentile of the NC, suggesting a high burden of cells with critically short TL in the patients with MDS.

Few studies have analyzed the association of mutation status; only that of the telomerase complex-related genes and TL in MDS patients [17, 18]. Some patients with telomere attrition had *TERC* or *TERT* mutations, but only a small number of patients were included in each study [17]. Therefore, we evaluated TL in relation to mutation of myeloid neoplasia-related genes and genes commonly found in hematologic malignancies. Patients without gene mutations had shorter TL than those with mutation (regardless of the gene), but comparing the TL between those and those without each specific gene mutation, the difference was only present for *CSF3R* gene. In the present study, the population with TL below the 10th percentile TL of the NC was markedly low in patients with *CSF3R* mutation. It is well known that mutation of *CSF3R* is accompanied in congenital neutropenia. The congenital neutropenia patients that did not develop leukemia had an incidence of *CSF3R* mutation in 34 %, compared to 78 % for patients who showed transformation into acute leukemia [41]. Mutation of *CSF3R* could confer the proliferative activity to the hematopoietic stem cell, which possibly leads to a transformation to AML. This may be reflected in the TL of patients with *CSF3R*, having lesser portion of cells with short telomeres and one of our patients showed AML transformation shortly after diagnosis. The mutation site of *CSF3R* in MDS patients was different from the known site of mutation in congenital neutropenia but two of the patients showed *CSF3R* mutation reported in myeloid neoplasms such as chronic neutrophilic leukemia or chronic myelomonocytic leukemia [42].

The hypothesis of this study was that TL erosion might be more significant in certain groups of patients with gene mutations that are known be correlated with telomere dysfunction such as *SRSF2* [16]. However, as TL is known to be affected by many other factors including age, environment, gender, stress, oxidative stress, and emotion [20, 43, 44], we were unable to show a correlation of TL in relation to a single gene mutation except for CSF3R. Contrary to our expectation, we found out that regardless of the gene mutation type, those patients with more than one gene mutation had higher TL than those without mutation. This finding somewhat contrasts with the recently reported result of aplastic anemia patients. Dumitriu et al. reported that telomere attrition and somatic mutations precede monosomy 7 and that the accumulation of short telomere in each chromosome may have a link to the development of aneuploidy in severe aplastic anemia (AA) [45]. This explanation can be applied to MDS evolved from previous AA. However, we infer that telomere attrition in AA exert a different influence on hematopoietic cells in case of MDS. AA has a decreased hematopoietic stem cell pool, but MDS has a dysfunctional hematopoietic stem cell pool, which has already acquired oncogenic mutations. Although the patients with gene mutations had longer TL than those without mutation in this study, the TL was still shorter than those of the NC. Comparisons within MDS groups may be less useful than the comparison of TL within an individual since telomere lengths are affected by many factors [20, 43, 44] including therapy, which was shown in a study that evaluated TL changes with treatment [46]. Another explanation may be that the mutations found in these patients were in the genes related to splicing machinery, DNA methylation, chromatin modification, or cohesion complexes and they are known to be mutated in MDS patients as driver/oncogenic mutations [14]. Therefore, these oncogenic mutations may have given the MDS cells the characteristics of “cancer” cells, which maintain telomere lengths [47], thus showing longer TL in patients with gene mutation. On the other hand, another explanation may be that these patients without gene mutations may have incorporated a mechanism that further shortens the length of the telomeres in this group. Mutations in other genes not included in this study such as *TERC* or *TERT* may give clue to these patients [17, 18]. Since no other study compared the TL of MDS patients and the presence of genetic mutation commonly found in hematologic malignancies, further studies would help elucidate the relationship between the telomere length and somatic mutations.

In addition, as TL is also regulated by the telomerase activity (TA) [1], we believe that the TA results would give additional information about the telomere dynamics. However, due to the sample limitation, TA could not be measured. TA was measured in MDS in few other studies, and MDS showed normal to low levels of telomerase activity [9, 48] despite the short TL. These results suggest the disruption of telomere maintenance in patients with MDS and suggest the maintenance of TL may be to some extent independent of TA incorporating alternative lengthening of telomere mechanisms, as is found in 10–15 % of the tumors [47].

The evaluation of TL with disease progression may be more informative and some reported telomere attrition during progression to acute myeloid leukemia [38, 49] but were shown in a small number of patients, thus requiring further study. In our study, there was no correlation of TL with various clinical subgroups and cytogenetic abnormalities, which is in line with previous studies showing inconsistent association between clinical factors and TL [9, 12]. Our finding may partly be due to the small number of patients included in our study, which is a major limitation of this study. Since cells with critically shortened telomeres may show repeated fusion bridge cycles, which leads to accumulation of chromosomal abnormalities, the relationship between chromosomal aberrations [50] and TL were also assessed. However, no significant change was found in relation to those with or without chromosomal aberrations. In addition to *TP53* mutation, *U2AF1* mutation was also suggested to be associated with a poor risk [51, 52] and was incorporated in the prognostic model along with the blast count and having high percentage of cells (≥80 %) with TL lower than the 10th percentile of the NC. Although the shortest telomere, the minimum TL, which is thought to be critical in the maintenance of cell survival, did not show prognostic significance in our study, we have found that having a high percentage of cells with short TL compared to NC may be of prognostic significance. The short TL was defined by having shorter than the lower 10th percentile value of the NC and this value may already suggest a portion of cells with critically short telomeres. Despite the limitation that Q-FISH measures the relative TL, the comparison made with the NC allowed us to confirm the shortened TL in MDS. Measuring TL in individual cells by Q-FISH also enabled us to identify the portion of cells with the short telomeres, which is not obtainable when measured by Southern blot or QPCR, which can only show the average TL. Moreover, our model including the TL parameter was comparable in the prediction of survival by Harrell’s C-index suggesting a probability of incorporation of TL and somatic mutation as a useful marker for prognostication.

## Conclusions

In summary, we evaluated the telomere lengths of MDS patients at a single-cell level by quantitative FISH assessing the shortest TL in each individual along with the average and distribution of TLs. Patients with myelodysplastic syndrome showed short telomere lengths compared to the control with a significant proportion of cells having shorter telomeres than the 10th percentile of the normal control. A proportion of cell population with shorter TL below the 10th percentile of the normal control was significantly larger. We conclude that MDS is characterized by concentrated hematopoietic cells with short telomere, a kind of senescent cells. There were no specific somatic mutation or cytogenetic aberration correlating with short TL, but patients without somatic mutation harbored short telomere than those with somatic mutation. Interestingly, *CSF3R* mutation correlated with longer telomere length, and proportion of short telomere length was significantly small. TL parameter showed independent prognostic significance and incorporating somatic mutation and TL in addition to known prognostic markers may give additional prediction power for prognosis.

## Abbreviations

AML, acute myeloid leukemia; BM, bone marrow; FISH, fluorescence in situ hybridization; IPSS, International Prognostic Scoring System; NC, normal control; MDS, myelodysplastic syndrome; OS, overall survival; SD, standard distribution; T/C, telomere to centromere; TL, telomere length; WHO, World Health Organization

## Additional file

Additional file 1: Supplementary method. Figure S1. Correlation of gene categories and cytogenetic abnormalities. Table S1. Genes included in the target sequencing and its gene categories. Table S2. Telomere lengths according to WHO categories or IPSS subgroups. Table S3. Telomere lengths according to laboratory results. Table S4. Telomere lengths according to mutations in gene groups. (DOC 803 kb)
