# Supplementary material for: Short telomere length and its correlation with gene mutations in myelodysplastic syndrome
Source: J Hematol Oncol. 2016 Jul 28;9:62. doi: 10.1186/s13045-016-0287-9 (PMC4964031; doi:10.1186/s13045-016-0287-9)
Supplement: Additional file 1: — Supplementary method. Figure S1. Correlation of gene categories and cytogenetic abnormalities. Table S1. Genes included in the target sequencing and its gene categories. Table S2. Telomere lengths according to WHO categories or IPSS subgroups. Table S3. Telomere lengths according to laboratory results. Table S4. Telomere lengths according to mutations in gene groups. (DOC 803 kb) [file 13045_2016_287_MOESM1_ESM.doc]

**Supplementary Method**

Summary of data-processing pipeline algorithm used for variant calling of somatic mutations is as follows: FASTQ files from HiSeq 2500 (Illumina, San Diego, CA, USA) were processed and aligned to the human reference genome 19 using Burrows-Wheeler Aligner (Li & Durbin, 2010) under the default parameters. Duplicate reads were removed using Picard MarkDuplicates (Broad Institute, <http://broadinstitute.github.io/picard>). Low quality data was eliminated by eliminating reads with less than 10 mapping quality and less than 20 base quality. Single nucleotide variations (SNVs) and indels were sorted. Among the variants found, low quality data was once more eliminated by mapping quality and allele frequency. Synonymous variants were removed. Those with allele frequency ≥ 0.005 in 1000 Genomes, NHLBI Exome Sequencing Project (ESP6500) and those with ≥ 0.002 allele frequency in in-house Korean exome database were removed. The candidate mutations registered in COSMIC v60 database (<http://www.sanger.ac.uk/genetics/CGP/cosmic>) was rescued and the ambiguous missense SNVs predicted to be benign by PolyPhen (Adzhubei *et al,* 2010) was removed .

**References**

Adzhubei IA, Schmidt S, Peshkin L, Ramensky VE, Gerasimova A, Bork P, Kondrashov AS, Sunyaev SR. (2010) A method and server for predicting damaging missense mutations. *Nature Methods*, **7**, 248-249.

Li H. & Durbin R. (2010) Fast and accurate long-read alignment with Burrows-Wheeler Transform. *Bioinformatics*, **26**, 589-595.

**Figure S1.** Correlation of gene categories and cytogenetic abnormalities.


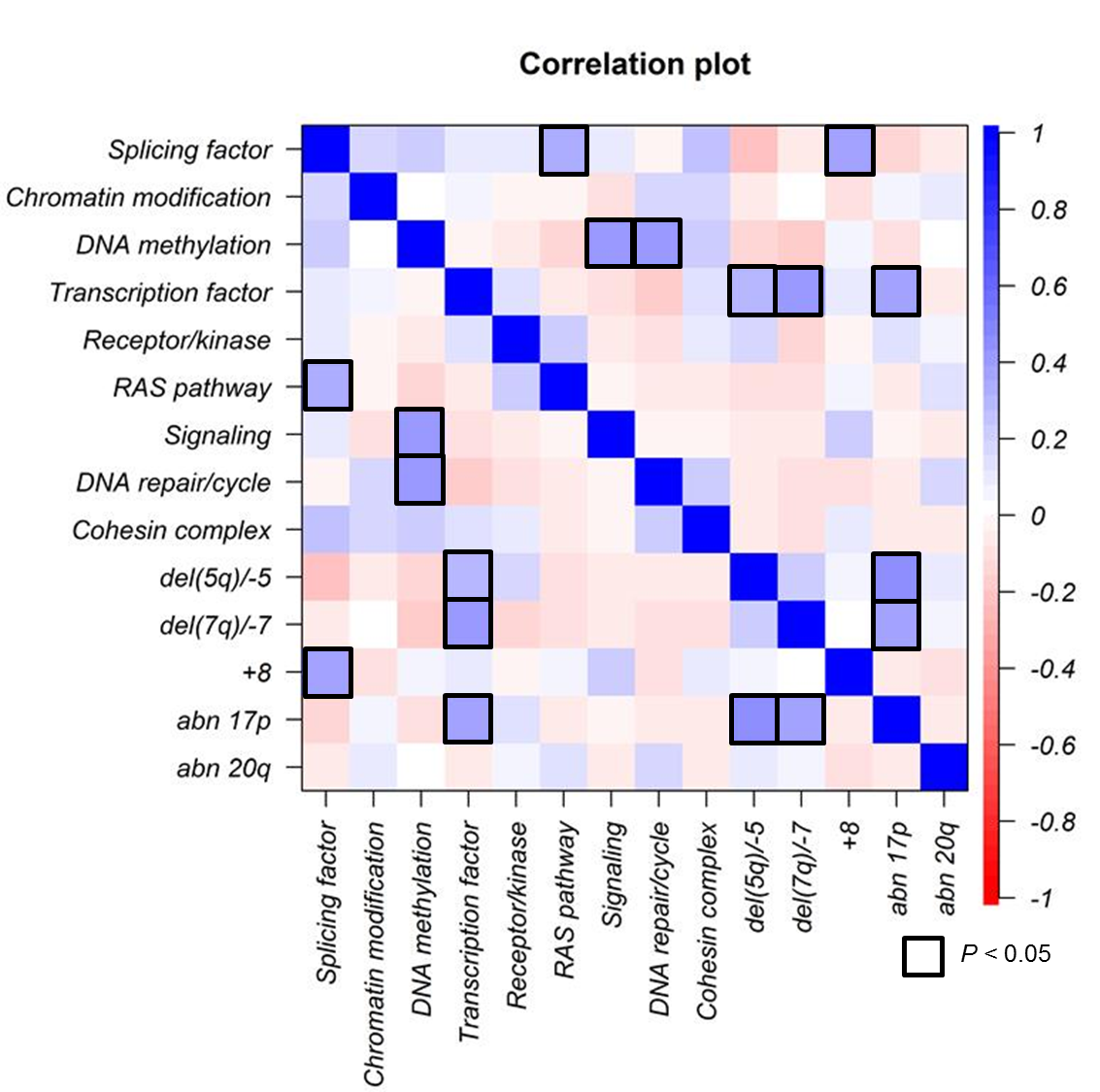


| **Table S1**. Genes included in the target sequencing and its gene categories | | | | | | | |  |
| --- | --- | --- | --- | --- | --- | --- | --- | --- |
| Gene categories | Genes |  |  |  |  |  |  |  |
| Splicing machinery | *PRPF40B* | *SF1* | *SF3A1* | *SF3B1* | *SRSF2* | *U2AF1* | *U2AF2* | *ZRSR2* |
| DNA methylation | *DNMT3A* | *IDH1* | *IDH2* | *TET2* |  |  |  |  |
| Chromatin modification | *ASXL1* | *ATRX* | *EZH2* | *BRD4* |  |  |  |  |
| Transcription factor | *BCOR* | *BRD2* | *CEBPA* | *EGR2* | *ETV6* | *GATA1* | *GATA2* | *IKZF1* |
|  | *NPM1* | *PHF6* | *RUNX1* | *TCF12* | *TP53* | *WT1* |  |  |
| Receptor/Kinases | *BIRC3* | *CSF1R* | *CSF3R* | *FBXW7* | *FLT3* | *JAK2* | *KIT* | *MPL* |
|  | *NOTCH1* |  |  |  |  |  |  |  |
| RAS pathway | *BRAF* | *CBL* | *KRAS* | *NF1* | *NRAS* | *PTPN11* |  |  |
| Cell signaling | *ITPKB* | *MAPK1* | *MYD88* | *PRKD3* | *SH2B3* |  |  |  |
| DNA repair/Cell cycle | *ATM* | *BARD1* | *BRCC3* | *CCND1* | *CDKN2A* | *RB1* |  |  |
| Cohesin | *RAD21* | *SMC1A* | *SMC3* | *STAG2* |  |  |  |  |
| Miscellaneous | *CARD6* | *CHD2* | *DAP3* | *DDX3X* | *DIS3* | *EEF1E1* | *FAM46C* | *FAT4* |
|  | *HIST1H1E* | *KIAA0355* | *KLHL6* | *LAMB4* | *LRP1B* | *MED12* | *NFKBIE* | *PLEKHG5* |
|  | *POLG* | *POT1* | *PTEN* | *RIPK1* | *SAMHD1* | *SCRIB* | *SETBP1* | *SMARCA2* |
|  | *TGM7* | *XPO1* | *ZMYM3* |  |  |  |  |  |

| **Table S2.** Telomere lengths according to WHO categories or IPSS subgroups | | | | | | | | |  |  |  |  |  |  |  |
| --- | --- | --- | --- | --- | --- | --- | --- | --- | --- | --- | --- | --- | --- | --- | --- |
| Clinical subgroups | N | Telomere Lengths (T/C ratio) | | | | | | | | | | | | | |
|  |  | Minimum | *P-*value | Q1 | *P-*value | Median | *P-*value | Average | *P-*value | Average of 0-10 percentile | *P-*value | Standard deviation | *P-*value | Cells under 10 percentile of normal control (%) | *P-*value |
| WHO categories | |  |  |  |  |  |  |  |  |  |  |  |  |  |  |
| RCUD | 12 | 1.79 ± 1.48 | 0.364 | 5.36 ± 2.68 | 0.654 | 7.76 ± 3.35 | 0.612 | 9.04 ± 3.85 | 0.505 | 2.88 ± 1.71 | 0.473 | 5.64 ± 2.93 | 0.246 | 52.0 ± 26.6 | 0.470 |
| RARS | 4 | 1.30 ± 0.93 |  | 3.31 ± 2.04 |  | 5.76 ± 2.89 |  | 6.84 ± 2.84 |  | 1.73 ± 1.09 |  | 4.74 ± 1.36 |  | 69.2 ± 24.5 |  |
| RCMD | 13 | 2.43 ± 2.01 |  | 6.23 ± 3.74 |  | 9.53± 5.63 |  | 11.06 ± 5.64 |  | 3.30 ± 2.06 |  | 6.98 ± 3.06 |  | 52.8 ± 25.8 |  |
| RAEB | 20 | 1.80 ± 1.28 |  | 5.66 ± 3.30 |  | 9.00 ± 4.59 |  | 10.73 ± 4.87 |  | 2.98 ± 1.91 |  | 7.49 ± 2.89 |  | 46.1 ± 25.9 |  |
| MDS, U | 9 | 2.20 ± 1.20 |  | 5.20 ± 2.13 |  | 8.55 ± 4.31 |  | 10.06 ± 4.74 |  | 2.99 ±1.32 |  | 6.66 ± 3.63 |  | 57.2 ± 19.2 |  |
| Revised IPSS subgroups | |  |  |  |  |  |  |  |  |  |  |  |  |  |  |
| Very Low | 4 | 1.89 ± 0.96 | 0.795 | 4.36 ± 1.00 | 0.865 | 6.66 ± 2.06 | 0.662 | 7.70 ± 1.84 | 0.504 | 2.65 ± 0.84 | 0.871 | 4.54 ± 0.69 | 0.187 | 57.6 ± 16.8 | 0.881 |
| Low | 14 | 1.68 ± 1.12 |  | 4.78 ± 1.81 |  | 7.15 ± 2.53 |  | 8.46 ± 3.12 |  | 2.62 ±1.16 |  | 5.62 ± 2.72 |  | 57.2 ± 22.8 |  |
| Intermediate | 14 | 2.22 ± 2.09 |  | 6.28 ± 3.94 |  | 9.85 ± 5.51 |  | 11.52 ± 5.49 |  | 3.21 ± 2.24 |  | 7.46 ± 3.04 |  | 50.3 ± 25.9 |  |
| High | 14 | 2.28 ± 1.55 |  | 6.27 ± 3.72 |  | 9.73 ± 5.74 |  | 11.30 ± 6.17 |  | 3.49 ± 2.13 |  | 7.26 ± 3.68 |  | 50.0 ± 31.5 |  |
| Very High | 12 | 1.68 ± 1.04 |  | 4.88 ± 2.49 |  | 8.01 ± 3.34 |  | 9.67 ± 3.60 |  | 2.59 ± 1.48 |  | 7.04 ± 2.64 |  | 49.1 ± 22.1 |  |
| Total | 58 | 1.97 ± 1.47 |  | 5.49 ± 3.05 |  | 8.57 ± 4.45 |  | 10.08 ± 4.73 |  | 2.95 ± 1.76 |  | 6.67 ± 3.02 |  | 52.1 ± 24.9 |  |

| **Table S3.** Telomere lengths according to laboratory results | | | | | | | |  |  |  |  |  |  |  |  |
| --- | --- | --- | --- | --- | --- | --- | --- | --- | --- | --- | --- | --- | --- | --- | --- |
| Laboratory parameters | N | Telomere Lengths (T/C ratio) | | | | | | | | | | | | | |
|  |  | Min | *P-*value | Q1 | *P-*value | Median | *P-*value | Average | *P-*value | Average of 0-10 percentile | *P-*value | Standard deviation | *P-*value | Cells under 10 percentile of normal control (%) | *P-*value |
| Hemoglobin (g/dL) | |  |  |  |  |  |  |  |  |  |  |  |  |  |  |
| < 8 | 29 | 1.95 ± 1.63 | 0.669 | 4.92 ± 2.81 | 0.222 | 7.56 ± 4.02 | 0.098 | 9.21 ± 4.61 | 0.114 | 2.74 ± 1.66 | 0.405 | 6.32 ± 3.24 | 0.287 | 59.9 ± 22.5 | 0.020 |
| ≥ 8 | 29 | 1.99 ± 1.33 |  | 6.06 ± 3.22 |  | 9.58 ± 4.71 |  | 10.96 ± 4.77 |  | 3.15 ± 1.86 |  | 7.02 ± 2.78 |  | 44.3 ± 25.2 |  |
| Absolute neutrophil count (/µL) | | |  |  |  |  |  |  |  |  |  |  |  |  |  |
| < 1000 | 25 | 1.94 ± 1.77 | 0.319 | 5.35 ± 3.48 | 0.495 | 8.45 ± 4.87 | 0.712 | 10.15 ± 5.59 | 0.783 | 2.87 ± 1.97 | 0.375 | 6.99 ± 3.56 | 0.869 | 58.4 ± 26.3 | 0.104 |
| ≥ 1000 | 33 | 1.99 ± 1.23 |  | 5.60 ± 2.72 |  | 8.65 ± 4.18 |  | 10.03 ± 4.06 |  | 3.01 ± 1.62 |  | 6.43 ± 2.56 |  | 47.4 ± 23.0 |  |
| Platelet count (10^3^/µL) | | |  |  |  |  |  |  |  |  |  |  |  |  |  |
| < 50 | 17 | 1.78 ± 1.06 | 0.811 | 4.46 ± 2.31 | 0.086 | 7.33 ± 3.89 | 0.174 | 8.71 ± 4.09 | 0.135 | 2.53 ± 1.45 | 0.301 | 6.06 ± 3.05 | 0.301 | 60.8 ± 23.0 | 0.086 |
| ≥ 50 | 41 | 2.04 ± 1.62 |  | 5.92 ± 3.24 |  | 9.08 ± 4.62 |  | 10.65 ± 4.91 |  | 3.12 ± 1.87 |  | 6.92 ± 3.00 |  | 48.5 ± 25.1 |  |
| Bone marrow blast count (%) | | |  |  |  |  |  |  |  |  |  |  |  |  |  |
| < 5 | 40 | 2.05 ± 1.57 | 0.551 | 5.55 ± 3.17 | 0.966 | 8.58 ± 4.66 | 0.775 | 10.07 ± 5.02 | 0.724 | 2.98 ± 1.77 | 0.712 | 6.53 ± 3.22 | 0.338 | 54.1 ± 24.7 | 0.373 |
| ≥ 5 | 18 | 1.78 ± 1.27 |  | 5.37 ± 2.85 |  | 8.53 ± 4.10 |  | 10.12 ± 4.17 |  | 2.89 ± 1.80 |  | 6.99 ± 2.57 |  | 69.9 ± 25.7 |  |
| Bone marrow fibrosis | | |  |  |  |  |  |  |  |  |  |  |  |  |  |
| Present | 4 | 1.51 ± 1.32 | 0.431 | 4.19 ± 3.49 | 0.200 | 6.30 ± 5.21 | 0.150 | 7.37 ± 5.33 | 0.141 | 2.41 ± 2.09 | 0.332 | 4.67 ± 2.55 | 0.141 | 66.8 ± 34.5 | 0.223 |
| Absent | 54 | 2.00 ± 1.49 |  | 5.59 ± 3.02 |  | 8.74 ± 4.40 |  | 10.28 ± 4.68 |  | 2.98 ± 1.75 |  | 6.82 ± 3.02 |  | 51.0 ± 24.1 |  |
| Cellularity |  |  |  |  |  |  |  |  |  |  |  |  |  |  |  |
| < 25% | 5 | 1.27 ± 0.44 | 0.309 | 4.87 ± 1.20 | 0.989 | 7.82 ± 1.98 | 0.966 | 9.14 ± 2.36 | 0.854 | 2.18 ± 0.67 | 0.367 | 6.12 ± 2.56 | 0.599 | 49.3 ± 16.7 | 0.831 |
| ≥ 25% | 50 | 2.07 ± 1.55 |  | 5.63 ± 3.23 |  | 8.76 ± 4.73 |  | 10.31 ± 5.00 |  | 3.07 ± 1.85 |  | 6.81 ± 3.14 |  | 51.6 ± 25.9 |  |

| **Table S4**. Telomere lengths according to mutations in gene groups | | | | | | | |  | |  | |  | |  | |  | |  |  | |  |  | |  |  | |
| --- | --- | --- | --- | --- | --- | --- | --- | --- | --- | --- | --- | --- | --- | --- | --- | --- | --- | --- | --- | --- | --- | --- | --- | --- | --- | --- |
| Gene or cytogenetic changes | | N | Mean age | P-value | Telomere Lengths (T/C ratio) | | | | | | | | | | | | | | | | | | | | | |
|  |  |  |  |  | Minimum | *P-*value | Q1 | | *P-*value | | Median | | *P-*value | | Average | | *P-*value | Average of 0-10 percentile | | *P-*value | Standard deviation | | *P-*value | Cells under 10 percentile of normal control (%) | | *P-*value |
| Splicing | Mut(+) | 20 | 63.0 | 0.578 | 2.13 ± 1.32 | 0.239 | 6.02 ± 2.94 | | 0.159 | | 9.40 ± 4.56 | | 0.180 | | 10.98 ± 5.03 | | 0.232 | 3.26 ± 1.67 | | 0.169 | 7.28 ± 3.47 | | 0.423 | 46.9 ± 24.4 | | 0.220 |
|  | Mut(-) | 38 | 64.1 |  | 1.88 ± 1.56 |  | 5.22 ± 3.11 | |  | | 8.13 ± 4.39 | |  | | 9.61 ± 4.57 | |  | 2.78± 1.81 | |  | 6.35 ± 2.74 | |  | 54.9 ± 25.1 | |  |
| Chromatin modification | Mut(+) | 15 | 65.1 | 0.950 | 2.09 ± 2.15 | 0.483 | 5.16 ± 3.54 | | 0.389 | | 7.83 ± 4.51 | | 0.399 | | 9.20 ± 4.84 | | 0.351 | 2.90 ± 2.12 | | 0.664 | 5.73 ± 2.25 | | 0.245 | 57.3 ± 22.7 | | 0.389 |
|  | Mut(-) | 43 | 63.2 |  | 1.93 ± 1.18 |  | 5.61 ± 2.90 | |  | | 8.83 ± 4.46 | |  | | 10.39 ± 4.71 | |  | 2.96 ± 1.65 | |  | 7.00 ± 3.20 | |  | 50.3 ± 25.6 | |  |
| DNA methylation | Mut(+) | 11 | 72.9 | 0.008 | 2.52 ± 1.71 | 0.145 | 5.99 ± 3.01 | | 0.445 | | 9.03 ± 3.91 | | 0.445 | | 10.67 ± 4.00 | | 0.388 | 3.50 ± 1.92 | | 0.201 | 7.05 ± 3.11 | | 0.627 | 42.5 ± 23.3 | | 0.162 |
|  | Mut(-) | 47 | 61.5 |  | 1.84 ± 1.40 |  | 5.38 ± 3.08 | |  | | 8.46 ± 4.60 | |  | | 9.94 ± 4.92 | |  | 2.82 ± 1.72 | |  | 6.58 ± 3.02 | |  | 54.4 ± 25.0 | |  |
| Transcription factor | Mut(+) | 18 | 62.9 | 0.454 | 1.92 ± 0.99 | 0.614 | 5.11 ± 2.19 | | 0.847 | | 8.57 ± 3.51 | | 0.420 | | 10.37± 3.66 | | 0.282 | 2.83 ± 1.29 | | 0.650 | 7.56 ± 2.81 | | 0.039 | 49.2 ± 20.0 | | 0.401 |
|  | Mut(-) | 40 | 64.0 |  | 1.99 ± 1.66 |  | 5.67 ± 3.38 | |  | | 8.57 ± 4.86 | |  | | 9.95 ± 5.18 | |  | 3.00 ± 1.95 | |  | 6.27 ± 3.06 | |  | 53.4 ± 26.9 | |  |
| Receptor/  Kinases | Mut(+) | 9 | 65.1 | 0.739 | 1.96 ± 0.91 | 0.540 | 6.38 ± 3.76 | | 0.421 | | 9.74 ± 5.35 | | 0.433 | | 11.67 ± 5.87 | | 0.373 | 3.20 ± 1.98 | | 0.838 | 7.89 ± 3.03 | | 0.153 | 42.4 ± 26.7 | | 0.260 |
|  | Mut(-) | 49 | 63.4 |  | 1.97 ± 1.56 |  | 5.33 ± 2.92 | |  | | 8.35 ± 4.30 | |  | | 9.79 ± 4.51 | |  | 2.90 ± 1.74 | |  | 6.45 ± 3.00 | |  | 53.9 ± 24.4 | |  |
| RAS pathway | Mut(+) | 5 | 65.2 | 0.830 | 1.52 ± 0.87 | 0.687 | 5.29 ± 3.35 | | 0.788 | | 8.37 ± 5.17 | | 0.830 | | 9.74 ± 5.79 | | 0.667 | 2.59 ± 1.94 | | 0.433 | 6.49 ± 3.85 | | 0.687 | 52.3 ± 31.0 | | 0.979 |
|  | Mut(-) | 53 | 63.6 |  | 2.01 ± 1.52 |  | 5.51 ± 3.05 | |  | | 8.59 ± 4.44 | |  | | 10.11 ± 4.69 | |  | 2.98 ± 1.76 | |  | 6.69 ± 2.97 | |  | 52.9± 24.6 | |  |
| Signaling | Mut(+) | 2 | 72.5 | 0.392 | 1.21 ± 1.20 | 0.437 | 4.33± 2.53 | | 0.668 | | 6.39 ± 2.92 | | 0.559 | | 7.40 ± 3.64 | | 0.484 | 2.09 ± 1.39 | | 0.508 | 4.26 ± 1.71 | | 0.350 | 63.6 ± 30.6 | | 0.559 |
|  | Mut(-) | 56 | 63.4 |  | 2.00 ± 1.49 |  | 5.54 ± 3.07 | |  | | 8.65 ± 4.50 | |  | | 10.18 ± 4.76 | |  | 2.98 ± 1.78 | |  | 6.76 ± 3.06 | |  | 51.7 ± 24.9 | |  |
| DNArepair/  cycle | Mut(+) | 4 | 69.3 | 0.363 | 2.77 ± 1.99 | 0.248 | 6.49 ± 3.27 | | 0.467 | | 10.15 ± 4.92 | | 0.431 | | 12.00 ± 4.32 | | 0.248 | 3.75 ± 2.21 | | 0.302 | 8.04± 2.44 | | 0.348 | 38.8 ± 22.2 | | 0.274 |
|  | Mut(-) | 54 | 63.3 |  | 1.91 ± 1.44 |  | 5.42 ± 3.05 | |  | | 8.45 ± 4.45 | |  | | 9.94 ± 4.77 | |  | 2.89 ± 1.74 | |  | 6.57 ± 3.05 | |  | 53.2 ± 25.0 | |  |
| Cohesin | Mut(+) | 4 | 69.3 | 0.413 | 2.30 ± 2.20 | 0.988 | 5.41 ± 4.06 | | 0.668 | | 8.46 ± 6.01 | | 0.756 | | 9.95 ± 5.55 | | 0.871 | 3.20 ± 2.59 | | 0.871 | 6.93 ± 2.03 | | 0.689 | 53.7 ± 33.6 | | 0.779 |
|  | Mut(-) | 54 | 63.3 |  | 1.94 ± 1.43 |  | 5.50 ± 3.01 | |  | | 8.58 ± 4.39 | |  | | 10.09 ± 4.73 | |  | 2.93 ± 1.72 | |  | 6.65 ± 3.09 | |  | 52.0 ± 24.6 | |  |
| Miscellaneous | Mut(+) | 12 | 64.4 | 0.916 | 2.38 ± 2.38 | 0.954 | 5.62 ± 4.20 | | 0.502 | | 8.39 ± 5.34 | | 0.526 | | 9.92 ± 5.67 | | 0.591 | 3.19 ± 2.61 | | 0.591 | 6.28 ± 2.85 | | 0.673 | 57.6 ± 27.2 | | 0.266 |
|  | Mut(-) | 46 | 63.5 |  | 1.86 ± 1.14 |  | 5.46 ± 2.73 | |  | | 8.61 ± 4.26 | |  | | 10.13 ± 4.53 | |  | 2.88 ± 1.50 | |  | 6.78 ± 3.08 | |  | 50.7 ± 24.4 | |  |
